# The Effect of Particle Size on the Deposition of Solid Lipid Nanoparticles in Different Skin Layers: A Histological Study

**DOI:** 10.15171/apb.2016.06

**Published:** 2016-03-17

**Authors:** Zahra Mardhiah Adib, Saeed Ghanbarzadeh, Maryam Kouhsoltani, Ahmad Yari Khosroshahi, Hamed Hamishehkar

**Affiliations:** ^1^ Research Center for Pharmaceutical Nanotechnology and Students’ Research Committee, Tabriz University of Medical Sciences, Tabriz, Iran.; ^2^ Zanjan Pharmaceutical Nanotechnology Research Center, Department of Pharmaceutics, Faculty of Pharmacy, Zanjan University of Medical Sciences, Zanjan, Iran.; ^3^ Department of Oral and Maxillofacial Pathology, Faculty of Dentistry, Tabriz University of Medical Sciences, Tabriz, Iran.; ^4^ Biotechnology Research Center and Faculty of Pharmacy, Tabriz University of Medical Sciences, Tabriz, Iran.; ^5^ Drug Applied Research Center, Tabriz University of Medical Sciences, Tabriz, Iran.

**Keywords:** Solid lipid nanoparticle, SLN, Topical drug delivery, Skin penetration, Rhodamine B

## Abstract

***Purpose:*** In the present study the effect of particle size, as a substantial parameters in skin penetration, on the deposition depth and rate of SLNs in different layers of skin was explored.

***Methods:*** SLNs in different particle size ranges (80, 333 and 971 nm) made of Precirol as solid lipid were prepared using hot melt homogenization technique and pigmented by Rhodamine B to be able to be tracked in the skin under inspection of fluorescent microscopy. After 0.5 h, 3 h, 6 h and 24 h of SLNs administration on rat skin, animals were sacrificed and exercised skins were sliced by a freeze microtome. SLNs were monitored in the skin structure under fluorescence microscope.

***Results:*** The size of SLNs played a crucial role in the penetration to deep skin layers. The sub100 nm size range of SLNs showed the most promising skin penetration rate and depth mainly via hair follicles.

***Conclusion:*** The results of the present study indicated that the selection of an appropriate size of particles may be a valuable factor impacting the therapeutic outcomes of dermal drug administration.

## Introduction

Treating skin diseases topically has many advantages: it is a non-aggressive and easy way to deliver drug, it has fewer systemic side effects compared with parenteral or oral drug delivery routes, it is possible to use high drug concentrations on the skin, the frequency of application is reduced, especially for drugs with a short half-life, drug input is terminated at initial administration time points by removing the drug, and patient compliance is improved.^1,2^ The horny layer, or stratum corneum (SC), of the skin is the main barrier for drug delivery since only a small percentage of the applied drug can penetrate.^3^ To overcome low uptake rates, nano particulate systems are developed. Lipid based nanopraticles not only enhance skin absorption but also can allow drug targeting to the skin layers. Solid lipid nanoparticles (SLNs) are one of the main subclasses of lipid based nanocarriers which attracted major attention in dermal drug delivery.^4^ SLNs are colloidal carriers developed in 1991 as an alternative to traditional colloidal carriers, such as emulsions, liposomes, and polymeric micro- and nanoparticles, to overcome their drawbacks by protecting the incorporated drug against degradation and providing size stability during storage time, biocompatibility and biodegradability, respectively.^5^ Additionally, SLNs show many other advantages, such as control and target drug release, easy scale up, enhanced bioavailability of entrapped bioactive compounds, composition of physiological compounds or toxicologically-acceptable compounds, and avoidance of organic solvents.^6,7^ During the past few years, SLNs have been used for the topical application of various drugs, such as anti-inflammatory and antifungal drugs, isotretinoin, podophyllotoxin, vitamin A, and many others. SLNs also represent a promising carrier system for cosmetic active ingredients because of their numerous advantages over existing conventional formulations.^8-11^ There are different approaches to modulating the penetration of active ingredients into the skin. In general, increased penetration is desirable in cases of pharmaceutical purposes, either accumulation of drug in the upper skin layer for local treatment or to achieve permeation of the skin leading to systemic absorption. However, for cosmetic actives, penetration only to a limited degree is desired to prevent any pharmaceutical effects. Therefore, depending on the purpose of the topical formulation, different requirements might be fulfilled by dermal formulations.^12-14^ The degree to which active ingredients penetrate the skin from topically-applied formulations depends on many factors, e.g., the physicochemical properties of the active compound, the physical state of the SC, and the nature of the carrier (e.g., polarity of the solvent and particle size). It is assumed that a decrease in particle size will cause an increase in the amount of drug found in the deeper skin strata. The number of studies investigating how particle size on its own affects delivery and absorption of a drug substance into the skin is insufficient, and the results are controversial.^15-18^ The current study investigated and compared the influence of particle size on penetration depth of SLNs into different skin layers.

## Materials and Methods

### Materials

Precirol^®^ ATO5 was purchased from Gattefossé Company (Lyon, France). Span^®^ 80 was obtained from BDH Laboratory (Cambridge, England). Poloxamer^®^ 407 was supplied from *Sigma-*Aldrich Company (Darmstadt*, Germany*). Rhodamine B was obtained from Merck Chemicals Company (Darmstadt, Germany). Chloroform (Dae-Junge, Korea), methanol‏ (Caledon, Canada) and diethyl ether (KianKaveh Pharmaceutical and Chemical Company, Tehran, Iran) were used as received. All chemicals were pharmaceutical grade and used without further modification. All solutions were prepared with deionized water.

### Preparations of Rhodamine B-loaded SLN

SLNs were prepared by the hot melt homogenization method. The primary goal in the first step of SLN production is to prepare a nanoemulsion from molten solid lipid. Briefly, 2 g of solid lipid, Precirol was melted at about 70 °C and Span^®^ 80, as an oil phase surfactant, was added to the melted lipid phase. Subsequently, Rhodamine B was dissolved in water and added dropwise into the oil phase under stirring at different stirring rates by homogenizer (DIAX 900, Heidolph, Germany) for different time to prepare SLNs in different sizes (Table 1). Finally, aqueous phase (Poloxamer 3% w/v) was dropped into the oil phase, keeping the temperature at 70 °C under homogenization to prepare an oil in water nanoemulsion. Rhodamine B-loaded SLNs (0.004 %) were obtained by allowing the hot nanoemulsion to cool down at room temperature.^4^ The Rhodamine B aqueous solution (Poloxamer 3% w/v) in the same concentration of SLNs formulation dispersion was used as control. Formulations was used for skin penetration experiments within one month after preparation since our experiments showed size stability of SLNs at least for one month.

**Table 1:** Particle size distribution parameters of prepared solid lipid nanoparticles

**Formulation**	**Modal D (nm)**	MVD* (nm)	**D**_10%_ **(nm)	**D**_50%_**(nm)	**D**_90%_ ** **(nm)**
**SLN 1**	77	77	53	77	117
**SLN 2**	330	329	231	330	455
**SLN 3**	962	932	588	962	1628

*MVD: Mean Volume Diameter **D_10%_, D_50%_ and D_90%_ are the equivalent volume diameter at 10%, 50% and 90% cumulative volume, respectively.

### Characterization of Rhodamine B-loaded SLN

Particle size was analyzed by laser diffraction method using particle size analyzer (SALD 2101, Shimadzu, Japan) which provides information about the mean diameter of the bulk population based on the volume mean diameter (VMD). Prior to measurement, SLN formulation samples were diluted with double-distilled water. Each sample was measured in triplicate. Zeta potential of prepared SLN formulations were analyzed by a Malvern zeta analyzer (Nano ZS, Malvern, UK).^19,20^

The loading capacity percent (LC%) was expressed as the percentage of entrapped Rhodamine B to the lipid. The amount of loaded Rhodamine B was determined by first separation of the un-entrapped Rhodamine B by centrifugation method using of Amicon^®^ Ultra-15 with molecular weight cutoff of 100 kDa (Millipore, Germany) tube. The formulation was added to the upper chamber of the Amicon^®^ tube and then the tube was centrifuged (Sigma 3K30, Germany) at 5000 rpm for 5 minutes and then the concentration of free Rhodamine B in the lower chamber of Amicon^®^ tube was measured using UV/Visible spectrophotometer (Shimadzu-1601, Japan) (λ_max_ = 260 nm, linear in the range of 2-30 µg/mL with R2=0.998) and mathematically calculated according to the following equation:

$$LC\left(\%\right)=\frac{W_{\left(Encapsulated\;drug\right)}}{W_{\left(Total\;lipid\right)}}\times 100$$

W_(Encapsulated drug)_ is the amount of loaded Rhodamine B and W_(Total lipid)_ is the amount of used lipid in the preparation process. The formulations in the upper chamber of Amicon^®^ tube were rinsed five times by aqueous solution to eliminate unloaded Rhodamine B. These rinsed formulations were used for the rest experiments.^21,22^

### In vivo skin penetration study

Male Wistar rats (weighing 200–250 g) were obtained from Pasteur Institute (Tehran, Iran), housed in animal facilities of the Drug Applied Research Center (Tabriz University of Medical Science) and used for skin permeation and drug deposition studies. All animal experiments were conducted according to the Guide for Care and Use of Laboratory Animals of Tabriz University of Medical Sciences, Tabriz-Iran (National Institutes of Health Publication No 85-23, revised 1985). The abdominal full thickness skin was carefully and mildy shaved using an electric razor in a way to avoid stratum corneum damage. 200 µL of the each formulations with different size was applied on the skin surface and after 0.5, 3, 6 and 24 h exposure, rats were sacrified with excess ether inhalation and the exposed skin was cut into 1.5×1.5 cm^2^ pieces and rinsed with distilled water. Each set of experiments was performed in triplicate. Aqueous solution of Rhodamine B was also used as control. Skins were cut into vertical slices with 20 µm thickness by a freeze microtome and slices were stored at 4 ºC and analyzed within 24 h. The skin slices were investigated under both normal light and fluorescence microscopes. The images were taken from normal light and fluorescence microscope of the same area and the dye distribution in the skin was evaluated qualitatively.^4^

## Results and Discussion

**Figure 1:**
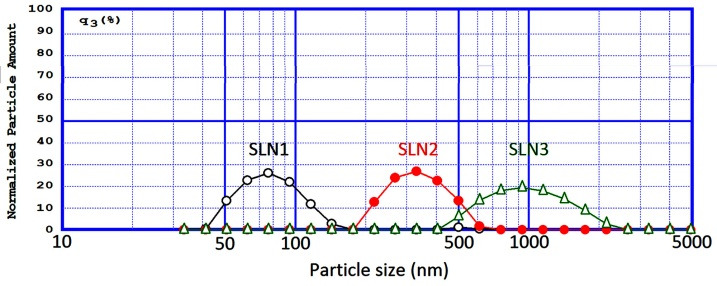
Particle size distribution pattern of SLN1, SLN2 and SLN3 formulations.

Rhodamine B was properly loaded into SLN formulations in LC percentages ranging 45% to 56%. 56%. Rhodamine B in higher and lower amounts than the previously investigated dose (0.004%) were prepared and applied to the skin. The concentrated formulations illustrated much more color intensity than the diluted ones which did not provide sufficient color strength to allow nanoparticle tracking (data not shown). The mean particle size of lipid nanoparticles usually depends on several factors, such as type and concentration of lipid surfactants and the viscosity in lipid phases.^4^ To avoid interfering with other effective parameters in skin penetration, the total amounts of lipid phase and surfactant were kept constant. The speed of homogenizer and the process time were changed to provide three SLN formulations with distinctly different submicron particle sizes. Table 1 illustrates the size characteristics of Rhodamine B-loaded SLN formulations. Figure 1 indicates that the fabricated SLN formulations had distinct size ranges which allowed for an accurate investigation of the impact of different submicron sizes on the skin penetration of SLNs.

**Figure 2:**
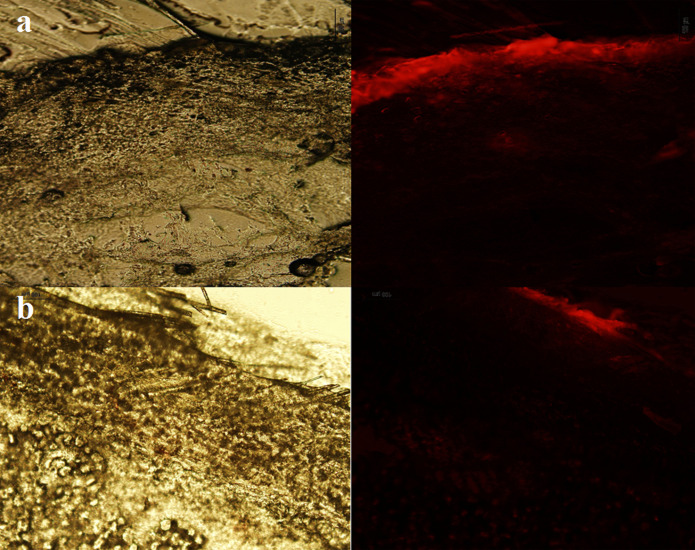
Fluorescence and normal light microscopes images of Rhodamin B solution 6 h (a) and (b) 24 h after application on rat skin.

Figure 2 shows the skin penetration capability of Rhodamine B solely in the solution state 0.5, 3, 6, and 24 h after administration onto the skin. The results indicated that, although the physicochemical characteristics of Rhodamine B (MW of 422 and Log P of 1.95) are suitable for skin penetration (MW<500 and Log P 1-4),^23,24^ no evidence of skin penetration by Rhodamin B was observed even after 6 and 24 h (Figure 2). A minor penetration of Rhodamine B solution may have occurred because of close contact with superficial junctions of the SC and furrows between the corneocytes islands and hair follicles, allowing the superficial spreading of active agents.^11,25^

**Figure 3:**
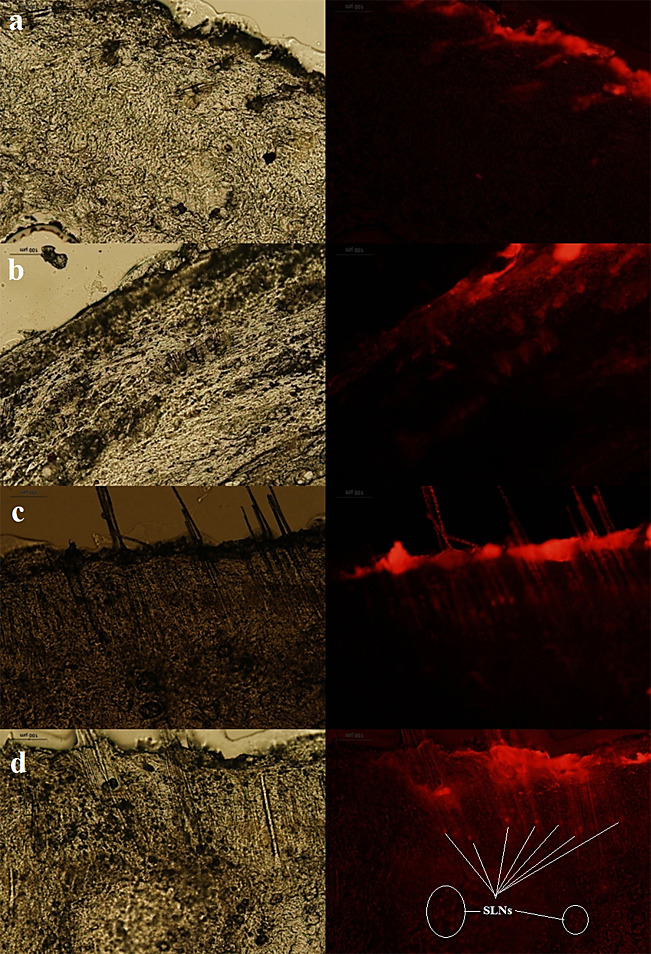
Fluorescence and normal light microscopes images of SLN1 formulation 0.5 h (a), 3 h (b), 6 h (c) and (d) 24 h after application on rat skin.

**Figure 4:**
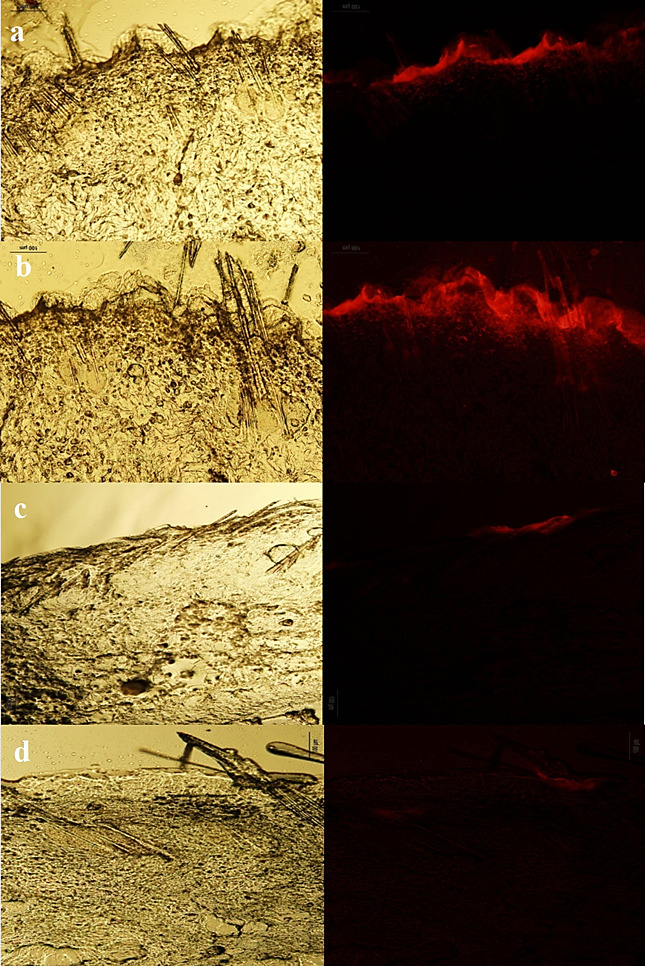
Fluorescence and normal light microscopes images of SLN2 formulation 0.5 h (a), 3 h (b), 6 h (c) and (d) 24 h after application on rat skin.

**Figure 5:**
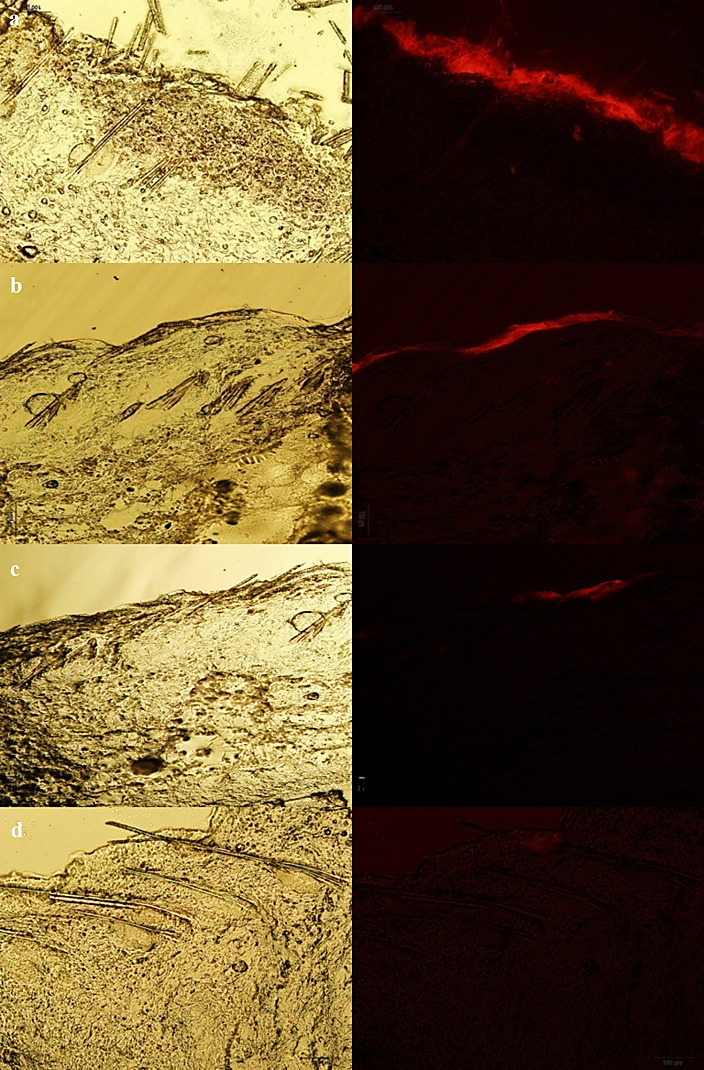
Fluorescence and normal light microscopes images of SLN3 formulation 0.5 h (a), 3 h (b), 6 h (c) and (d) 24 h after application on rat skin.

Figures 3, 4, and 5 show the normal light and fluorescence imaging microscopies of formulations SLN1, SLN2, and SLN3, respectively. To evaluate the rate of penetration, the distribution pattern of Rhodamine B was explored 0.5, 3, 6, and 24 h after the SLN formulation was administered onto the skin. The impact of SLN size on skin penetration was assessed by the results reported in Figures 3-5. The comparison of Figures 3a-5a indicated that only SLN1 could pass SC mainly through hair follicles (shown by circles in Figure 3a). Therefore, the sub-100 nm size range is crucial for the onset of action in dermal drug delivery. One characteristic that makes nanoparticles interesting for topical application is their tendency to diffuse and accumulate in hair follicles. The particles can aggregate in the follicular opening and penetrate along the follicular dug when applied onto the skin surface.^26,27^ It has been shown that nanoparticles preferentially permeate into the follicles, but not the SC, enabling high accumulation within the follicular reservoir.^17,28^ Correspondingly, the comparison of Figures 3c, d - 5c, d indicated that larger SLN particles (SLN2 and SLN3) could not penetrate into deep layers of skin even after 6 and 24 h. Only the SLN in the mid-size range (SLN2) could penetrate the upper skin layers (Figure 4b). SLN1 could penetrate into lower skin layers after 24 h (Figure 3d) as depicted by arrows. Figures 3b and 3d illustrate that hair follicles provided the most important and dominant penetration route of SLNs.

It has been reported that SLNs approximately 260 nm in size were able to penetrate into the deeper layers of the skin and preferentially accumulate in hair follicles.^16^ Other studies have also reported that hair follicles serve as long-term reservoirs for topically-applied drug substances, storing them for up to 10 days, which is ten times longer than the reservoir of the SC.^29^ The findings of the current study showed that sizes under 100 nm are in the appropriate size range for deep skin penetration. This result contradicts the report of Patzelt et al., which showed that reductions in particle size (643 nm, 470 nm, 300 nm, 122 nm) led to a significant reduction in particle penetration depth.^30^ In support of the current findings, Abddel Mottaleb et al. reported results indicating that the follicular penetration of nanoparticles increases following a decrease in size.^31^

## Conclusion

Skin is a widely-used route of delivery for various local and systemic drugs. It provides a natural physical barrier against particle penetration, but there are opportunities to deliver therapeutic nanoparticles. Most drug delivery particle technologies are based on lipid carriers, i.e. SLNs. It has already been shown for various drugs that topical formulations containing lipid nanoparticles can enhance penetration into the skin, thus increasing treatment efficiency, target the epidermis, and reduce systemic absorption and side effects. In the present study, the HMH method was used to prepare Rhodamine B-loaded SLNs with identical compositions and different submicron sizes. The findings concluded that sub-100 nm SLNs can efficiently penetrate into deep layers of skin mostly via hair follicles. In conclusion, nanoparticle size appears to be of major relevance for the enhancement of depth and rate of skin penetration by substances. The suitable mechanism for skin targeting still needs further study. Furthermore, a better understanding of how lipid nanoparticles modify drug penetration into the skin, how lipid particles interact with the lipids of the stratum corneum, and finally how they affect drug penetration is necessary.

## Acknowledgments

The authors would like to thank Drug Applied Research Center, Tabriz University of Medical Sciences for financial support. This article is based on a thesis submitted for Pharm D degree (No. 3773) in Faculty of Pharmacy, Tabriz University of Medical Sciences.

## Ethical Issues

Not applicable.

## Conflict of Interest

The authors report no conflicts of interest.
